# NRDR Inhibits the Migration of Endometrial Cancer Cells and Affects Their Gene Expression

**DOI:** 10.1155/sci5/2495655

**Published:** 2025-07-07

**Authors:** Ying Liu, Jiankai Shi, Shuxin Li, Shujun Liu, Yang Liu, Jiwei Liu, Xiaoying He, Chuncheng Liu, Libing Ma

**Affiliations:** Inner Mongolia Key Laboratory of Life Health and Bioinformatics, School of Life Science and Technology, Inner Mongolia University of Science & Technology, Baotou 014010, Inner Mongolia, China

**Keywords:** endometrial cancer, epithelial-mesenchymal transition, migration, NRDR

## Abstract

**Introduction:** Studying the pathogenesis of endometrial cancer is important to treatment of endometrial cancer. NADP(H)-dependent retinol dehydrogenase/reductase (NRDR) is associated with the development of cancer. Our previous research has found that NRDR can inhibit the synthesis of estradiol (E2) in granulosa cells. Based on the above statement, we speculate that NRDR may also be involved in the development of endometrial cancer. Therefore, this study investigated the expression patterns and mechanisms of NRDR in endometrial cancer.

**Material and Methods**: This study was performed using Ishikawa cells combined with multiple methods, including immunohistochemical staining, wound healing and Transwell migration assays, RNA-seq analysis, and so on.

**Results:** The results showed that NRDR was expressed in endometrial cancer tissues and uterine glands, and it was higher in endometrial cancer tissues of elderly patients. Wound healing assay and Transwell migration assay results showed that RNA interference targeting NRDR gene expression could promote the migration of endometrial cancer cells and the expression of α-SMA, Vimentin, and Twist. In addition, E2 could downregulate the expression of NRDR in endometrial cancer cells. Lastly, RNA-seq was performed on Ishikawa cells (RNA interference of NRDR), and the differentially expressed genes (DEGs) were analyzed. Further enrichment analysis of the functions and signaling pathways of DEGs using GO and KEGG revealed that DEGs were mainly enriched in intrinsic component of plasma membrane, integral components of plasma membrane and calcium signaling pathway.

**Conclusions**: In our study, it turns out that NRDR is a tumor suppressor in endometrial cancer cells. Through investigating the physiological function and molecular mechanism of NRDR in endometrial cancer, our experiment provides a theoretical basis for further understanding the pathogenesis of endometrial cancer.

## 1. Introduction

Uterine cancer is the most common gynecological malignancy in women, and endometrial cancer (EC) is the most common type of uterine cancer [1]. Women in their forties and fifties (usually postmenopausal) are most at risk for developing EC. Without prompt treatment, cancer, which originates from endometrial epithelial tissue, can spread to other organs and tissues in the body, including the ovaries [2–4]. Developing and researching diagnostic and therapeutic targets for EC is of significant importance [5]. In China, ovarian cancer has the highest incidence rate among gynecological malignancy patients, with EC coming in a close second [6]. EC incidence and mortality rates have steadily increased recently, particularly among young women, threatening most women's health [7]. EC has a younger age distribution, which will greatly impact health.

Retinoic acid metabolism plays an important role in promoting the normal differentiation and proliferation of cells and participates in immune regulation and anti-infection [8–10]. NADP(H)-dependent retinol dehydrogenase/reductase (NRDR) is an important metabolic enzyme involved in retinoic acid synthesis and metabolism. The *DHRS4* gene encodes NRDR, and this gene is a member of the short-chain dehydrogenase/reductase superfamily (SDR) [11]. The SDR superfamily is involved in the physiological synthesis of steroid hormones as well as the metabolism of vitamin A and carcinogens [12]. The NRDR protein has strong retinol oxidation and retinaldehyde reduction activities [13–15]. NRDR was first purified from rabbit liver by Professor Dongyang Huang and existed in other mammals, such as dogs, pigs, mice, and humans [16, 17]. In pigs with high androgen, NRDR is highly expressed in the testis [18]. Previous studies have found that NRDR is highly expressed in granulosa cells of the porcine ovary and plays a role in the synthesis of estradiol (E2) hormone in the granulosa cells of pigs [19]. Cervical cancer and other human cancers are also affected by NRDR [20]. NRDR promotes the proliferation and migration of the most common malignant primary brain tumor (glioma) [21]. The disorder of retinal and retinoic acid metabolism caused by abnormal splicing of NRDR and the functional disorder of NRDR may play a role in the development of cervical cancer [22].

Our primary objective of this study was to look at NRDR expression and associated functions in EC. It was found that NRDR is expressed in EC and that its expression is higher in the tissues of elderly patients with EC. We also found that NRDR could inhibit EC cell migration by activating the calcium signaling pathway. The NRDR function in EC provides new insight into this disease and identifies a potential therapeutic target.

## 2. Materials and Methods

### 2.1. Ethics

Informed consent (tissue microarray) was obtained from all subjects and/or their legal guardians and was approved by the Human Investigation Ethics Committee of Shanghai Outdo Biotech Company (Shanghai, China). All methods were performed in accordance with these guidelines and regulations. NMGKJDX Laboratory Animal Management Committee approval to carry out the study (Ethical Application Ref: NMGKJDX-2020-6).

### 2.2. Tissue Microarray

The EC Tissue Microarray (Product Number: HUteA060CS01) was provided by Shanghai Chip Screen Bioscience Co., Ltd. It contained 43 samples, including 34 EC tissue samples and 9 adjacent noncancerous tissue samples. The expression of NRDR in EC Tissue Microarray was assessed using immunohistochemistry.

### 2.3. Immunohistochemical Evaluation

Protein expression levels were assessed using a semiquantitative scoring system. Staining intensity was graded on a 4-point scale: 0 (negative), 1+ (weak), 2+ (moderate), and 3+ (strong). The percentage of positively stained cells was quantified across five randomly selected high-power fields (× 400 magnification). A composite score (range: 0–300) was calculated by multiplying the intensity score (0–3) by the positivity percentage (0%–100%). Based on receiver operating characteristic (ROC) curve analysis, specimens were stratified into low expression (composite score < 0.6) and high expression (≥ 0.6) groups to optimize clinical outcome discrimination. The associations between NRDR expression and categorical clinicopathological variables (age, grade, tumor size) were analyzed using the Pearson chi-square test. Statistical significance was defined as a two-tailed *p* < 0.05. All analyses were performed using GraphPad Prism version 9.0.

### 2.4. Cell Culture

The Ishikawa cells (human endometrial adenocarcinoma cell line) purchased from Hunan Fenghui Biotechnology Co., Ltd were used for the study. Its Research Resource Identifier (RRID) is RRID:CVCL_2529. Ishikawa cells were grown in DMEM (GIBCO) medium with 10% fetal bovine serum (FBS) and 1% penicillin/streptomycin (Invitrogen). Cells were incubated at 37°C and 5% CO_2_. All experiments were performed with mycoplasma-free cells.

### 2.5. Cells Transient Transfection and Treatment

NRDR-small interfering RNAs (siRNA) NRDR-siRNA1, NRDR-siRNA2, NRDR-siRNA3, and a negative control siRNA (NC-siRNA) were purchased from RiboBio (Guangzhou, China). Transient transfection procedures were performed as previously described [19]. We treated the cultured cells with retinol, retinal, TGFβ, and E2 for 0 (CON), 4, 8, 12, and 24 h, respectively. The samples were stored at −80°C before the followed experiments.

### 2.6. Wound-Healing Assay

Cells (1 × 10^5^ cells/mL) were seeded in 6-well plates and transfected for 48 h once they reached 50% confluences. The cell monolayer was scratched by the tip of a sterile 10 μL pipette, and the cells were cultured normally after the cell debris was cleaned with PBS. Then the wounded area was observed and photographed by an inverted fluorescence microscope (Nikon, Japan) at 0 h, 24 h, and 48 h. Using the Image J software, the wounded areas were measured by the width of the wounds.

### 2.7. Transwell Migration Assay

48 h after transfection with siRNA, cells were re-suspended in 200 μL DMEM medium (1 × 10^5^ cells/mL) and seeded into the upper chamber of the Transwell plate (8 μm pore size), while the lower chambers were added 700 μL of DMEM medium containing 15% FBS. After 20 h, cells were stained with crystal violet. The nonmigrated cells on the surface of the filter membrane were scrubbed before being recorded and photographed using an inverted fluorescence microscope (Nikon, Japan).

### 2.8. RNA Extraction and qRT-PCR

The total RNA of the cells (5 × 10^4^ cells/mL) was extracted in an RNase-free environment using RNAiso Plus reagent (Takara, Japan). The quality and concentration of the extracted RNA were detected by NanoDrop spectrophotometer, and qualified RNA was used for subsequent experiments. RNA was reverse transcribed into cDNA, according to the instructions of reverse transcription Kit (Takara, Japan). TB Green® Premix Ex Taq™ II (Tli RNaseH Plus) (Takara, Japan) and biosystems® 7500 real-time PCR system were used for qRT-PCR according to the instructions. Using GAPDH as internal reference gene, the relative expression of the gene was calculated by 2^−ΔΔCt^ method. The primer sequence of the genes was in Table S1.

### 2.9. Transcriptome Library Preparation and Sequencing

Transcriptome sequencing was performed by Shanghai Majorbio bio-pharma Biotechnology Co., LTD. (Shanghai, China) according to the manufacturer's instructions (Illumina, San Diego, CA). The transcriptome library uses 1 µg total RNA to isolate the messenger RNA using oligonucleotide (dT) beads and then fragment it. Then, the double-stranded cDNA was synthesized using the superscripted double-stranded cDNA synthesis kit (Invitrogen, CA) and random hexamer primers (Illumina), and the synthesized cDNA was end-repaired, phosphorylated, and “A” base added. A 300 bp cDNA target fragment library was selected on 2% Low Range Ultra agarose and 15 cycles were amplified with Phusion DNA polymerase (NEB). The peer RNA-seq library was sequenced using the Illumina NovaSeq 6000 sequencer and quantified using the TBS380 instrument.

### 2.10. Quality Control and Read Mapping

The experimental group and the control group each contain three biological replicates. The statistical power of this experimental design, calculated in RNASeqPower is 0.87. The “fastp” [23] with default parameters was used to trimmed and quality controlled for the raw paired-end reads. The clean reads were then aligned to the reference in orientation mode using the HISAT2 software [24]. The map read for each sample is assembled using StringTie [25].

### 2.11. Differential Expression Analysis and Functional Enrichment

The expression level of each gene is determined by transcription read this per million (TPM), at the same time RSEM (https://deweylab.biostat.wisc.edu/rsem/) is used to calculate gene abundance [26]. Analysis of gene differential expression between different groups was mainly carried out using Limma. Differentially expressed genes are defined by |log2(foldchange)| > 1 and *p*-adjust < 0.05 [27]. Further, DEGs were analyzed the function of enrichment by using the gene ontology (GO, https://www.geneontology.org) and kyoto encyclopedia of genes and genomes (KEGG). Goatools and KOBAS performed GO functional enrichment and KEGG pathway analysis, respectively (https://kobas.cbi.pku.edu.cn/home.do) [28].

### 2.12. Statistical Methods

Data were analyzed using GraphPad Prism 9.0 (GraphPad Software, San Diego, CA) and expressed as mean ± standard error of the mean (SEM). For comparisons between two groups, Student's *t*-test (two-tailed, unpaired) was applied. A *p* value < 0.05 was considered statistically significant.

## 3. Results

### 3.1. NRDR Was Expressed in EC Tissues and Uterine Gland

**Figure 1:**
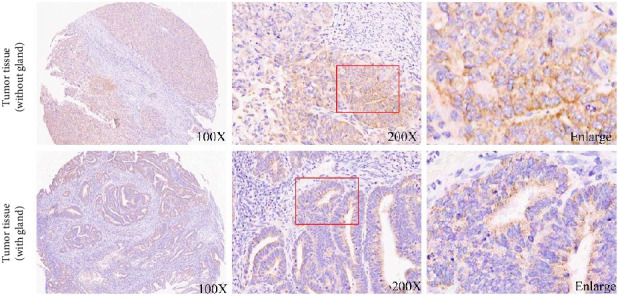
Expression of NRDR in endometrial cancer tissue. Expression of NRDR in endometrial cancer tissues detected using immunohistochemistry assay. The brown stain represents NRDR, while the blue represents nuclear counterstaining (DNA).

To investigate the role of NRDR in (EC), a tissue array was used to detect the protein expression of NRDR in EC. The samples included 34 cancerous tissues and 9 adjacent noncancerous tissues. The expression of NRDR was detected in EC tissues and uterine gland (Figure 1). Furthermore, the expression of NRDR was compared between cancerous tissues and adjacent noncancerous tissues. The findings revealed no significant differences between cancerous and adjacent noncancerous tissues (Table S2).

### 3.2. The expression of NRDR Was Increased in EC Tissues of Elderly Patients

The correlation between the expression of NRDR and the age of patients' grade, and tumor size of EC was analyzed, to learn more about its role in the disease's progression. The data demonstrated that NRDR expression is higher in the tissues of elderly patients with EC (*p*=0.028) (Table 1). However, the expression of NRDR had no direct correlation with tumor grade and tumor tissue size (Table 1).

### 3.3. NRDR Inhibited the Ability of Migration in EC Cells

**Figure 2:**
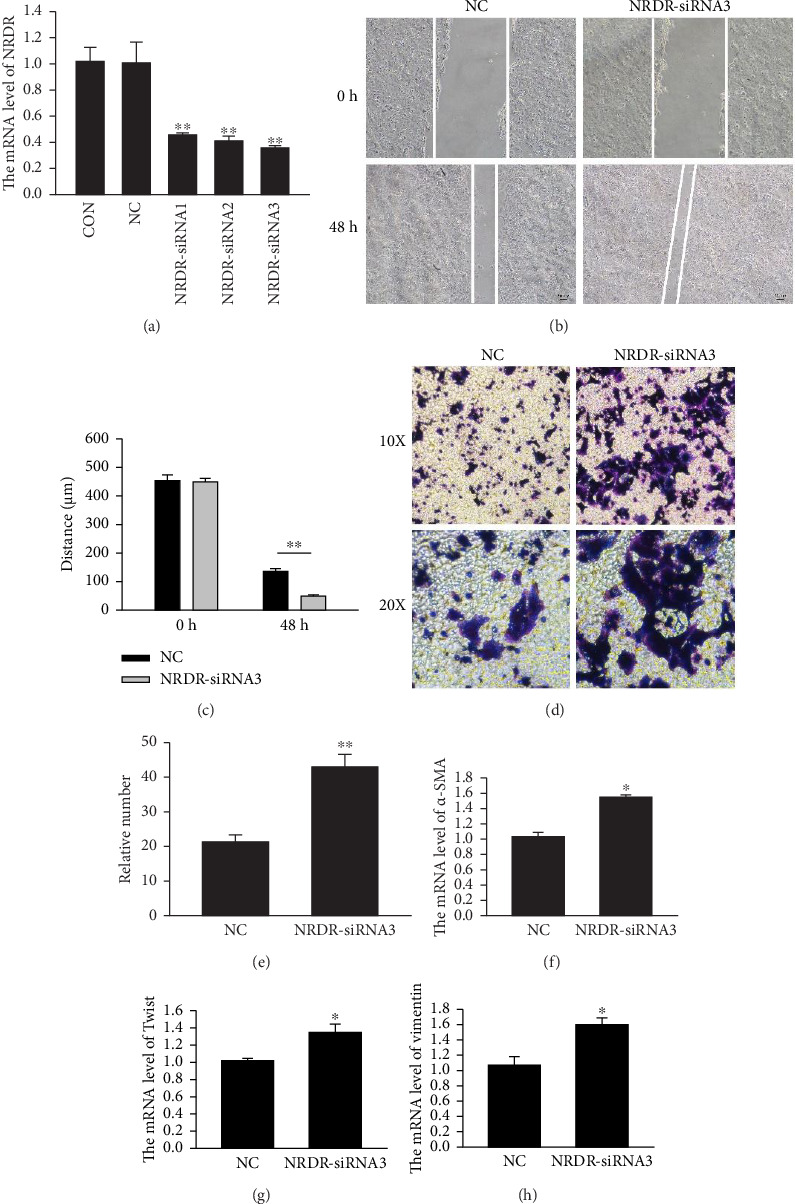
The role of NRDR in endometrial cancer cell migration. (a) The levels of NRDR mRNA in Ishikawa cells with different siRNAs treatment (*n* = 3). (b) Wound healing assay images showing migration of Ishikawa cells upon transfection with NRDR siRNAs and NC (Magnification, × 50). (c) The wound distances and statistical analysis of Ishikawa cell migration (*n* = 3). (d) The Transwell assay of Ishikawa cells after transfecting with NRDR siRNA (*n* = 3). (e) The relative number of migrated cells were counted and analyzed after transfecting with NRDR siRNA (*n* = 3). The values were expressed as the mean ± SEM of three independent experiments. The mRNA levels of a-SMA (f), Twist (g), and Vimentin (h) in Ishikawa cells by qRT-PCR, respectively. ^∗^*p* < 0.05, ^∗∗^*p* < 0.01. (*n* = 3).

Using NRDR siRNAs in cultured Ishikawa cells, the role of NRDR in EC cells was identified. First, qRT-PCR was used to confirm the siRNAs' efficiency to inhibit expression. Approximately 60% reduction in NRDR mRNA levels was observed 48 h after transfection in cultured cells when NRDR-siRNA2 and NRDR-siRNA3 were used (Figure 2(a)). The NRDR-siRNA3 with the highest inhibition efficiency was chosen for further testing. To explore the role of NRDR in EC cells, Ishikawa cells were transfected with NRDR-siRNA3 and NC-siRNA for 48 h. Afterward, EC migration was tracked using wound healing (Figures 2(b) and 2(c)) and Transwell assays (Figures 2(d) and 2(e)). Wound healing and Transwell assays showed that Ishikawa cells' migration abilities were significantly improved upon NRDR silencing with siRNA. This indicates that NRDR had an important role in migrating EC cells.

Epithelial mesenchymal transition (EMT) plays an important role in the metastasis of EC cells [29]. By inhibiting NRDR expression, we found that Ishikawa cells were more metastatic and invasive in wound-healing and Transwell assays. Furthermore, we found that NRDR-siRNA3 silencing changed the expression of EMT-related genes. EMT is defined by the upregulation of mesenchymal cell markers such as, α-SMA (Figure 2(f)), Twist (Figure 2(g)), and Vimentin (Figure 2(h)). Our results showed that the expressions of Vimentin, Twist, and α-SMA were significantly upregulated in NRDR knock-down Ishikawa cells.

### 3.4. Effects of Retinal, Retinol, TGFβ, and E2 on NRDR Expression in Ishikawa Cells

**Figure 3:**
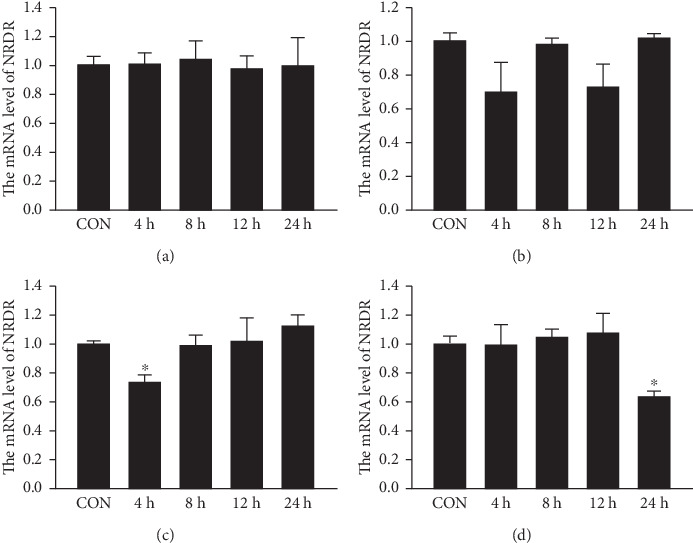
Effects of retinal, retinol, TGFβ, and E2 on NRDR expression in Ishikawa cells. The expression of NRDR after treatment with (a) retinol, (b) retinal, (c) TGFβ, and (d) E2. Results are shown as means ± SEM of three independent experiments and normalized with control, ^∗^*p* < 0.05.

NRDR is a key enzyme in retinol metabolism. So the Ishikawa cells were treated with 10 μM retinoic acid or retinol [30–32] for 0 (Control), 4, 8, 12, and 24 h, followed by quantification of NRDR expression. We found that retinoic acid or retinol did not affect the expression of NRDR in Ishikawa cells (Figures 3(a) and 3(b)). Similarly, studies showed that TGFβ and E2 are important in promoting EC progression [33, 34]. Ishikawa cells were treated with 10 ng/mL TGFβ [35], and 10 nM E2 [36], and the expression of NRDR was assessed. TGFβ had not affected in the expression of NRDR (Figure 3(c)). Approximately 40% of NRDR mRNA levels were downregulated by 24 h of E2 treatment (Figure 3(d)).

### 3.5. Transcriptome Sequencing of Ishikawa Cells

Ishikawa cells were transfected with NRDR-siRNA3 and NC for 48 h and collected for the transcriptome sequencing. To evaluate gene expression, transcriptome sequencing data was compared to the *Homo sapiens* gene database (https://asia.ensembl.org/Homo_sapiens/Info/Index). After removing adaptor sequences and low-quality reads, 901-755 million clean reads (paired-end 125 bp) were left (Table S3). With tophat2, around 95.30% of the clean reads were mapped to the *Homo sapiens* genome assembly 10.2 (range from 95.09% to 95.62%). A total of 58974 transcripts were identified across all samples.

**Figure 4:**
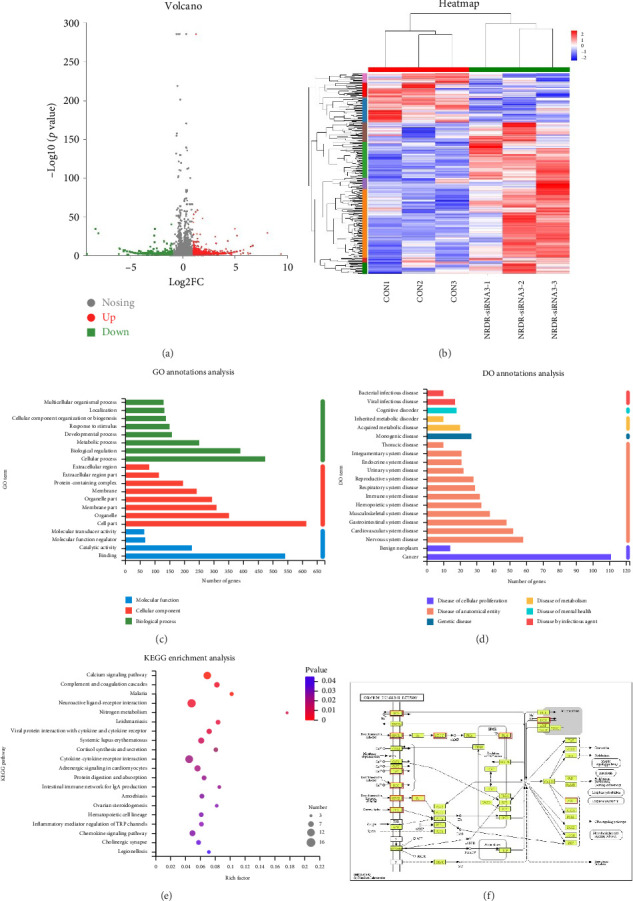
RNA-seq analysis. (a) The DEGs are displayed through volcano maps. Upregulated genes and downregulated genes were represented in red and green colors. (b) Heatmap of DEGs with hierarchical clustering between control and NRDR-siRNA3-treated Ishikawa cells (*n* = 3). (c, d) GO (c) and DO (d) enrichment analysis of DEGs showing pathways and processes that were enriched in NRDR-siRNA3 treatment as compared with NC in Ishikawa cells. Significant enrichment terms of DEGs were analyzed by Goatools (*p* < 0.05). (e) KEGG enrichment analysis of DEGs. KEGG enrichment analyses were based on KOBAS database (*p* < 0.05). (f) The DEGs in the calcium signaling pathway are shown, with red indicating upregulated genes.

We examined the effects of NRDR-siRNA3 on gene expression in EC cells. In the current study, a total of 1690 DEGs (1086 upregulated and 604 downregulated) were identified using the following criteria: *p*-adjust < 0.05 and |log2FC| > 1 (Figures 4(a) and 4(b)).

The DEGs were analyzed by GO and Disease Ontology (DO) according to their biological functions after NRDR inhibition in EC cells. The top 20 GO terms (8 biological processes, 8 cellular components, and 4 molecular functions) were strongly linked to malignant phenotypes (Figure 4(c)). At the molecular function level, aberrant activation of molecular transducers and enhanced binding activities promoted tumor proliferation and immune evasion. Key biological processes included metabolic reprogramming, immune system remodeling, and reactivation of developmental processes, synergistically driving metastasis. Cellular component analysis highlighted membrane receptors mediating oncogenic signaling, extracellular matrix degradation facilitating invasion, and organelles' dysfunction sustaining cancer cell survival. Furthermore, DO analysis associated these DEGs with bacterial/viral infections, inherited/acquired metabolic disorders, multisystem diseases, and cancers (Figure 4(d)).

Analysis results showed that DEGs were significantly enriched in top 20 KEGG-analyzed signaling pathways (Figure 4(e)). Pathway analysis revealed three interconnected functional clusters driving EC progression: (1) hormonal and metabolic reprogramming mediated by ovarian steroidogenesis, nitrogen metabolism, and cortisol synthesis, which collectively sustain estrogen-dependent proliferation and chemoresistance; (2) immune-inflammatory dysregulation involving cytokine-cytokine receptor interaction, systemic lupus erythematosus, intestinal immune network for IgA production, inflammatory mediator regulation of TRP channels, and Viral protein interaction with cytokine and cytokine receptor, indicative of chronic immunosuppression; and (3) metastatic competence driven by calcium signaling, chemokine signaling pathway, and so on. The first functional cluster was dominated by the calcium signaling pathway, which exhibited coordinated upregulation of key regulators including: SLC8A1, CHRM5, HTR6, CACNA1H, GRIN1, PARX2, PDGFRA, GNA14, ADCY4, PLN, NOS2, AKRA1B, and PTGFR (Figure 4(f)).

### 3.6. Validation of Changes in Gene Expression

**Figure 5:**
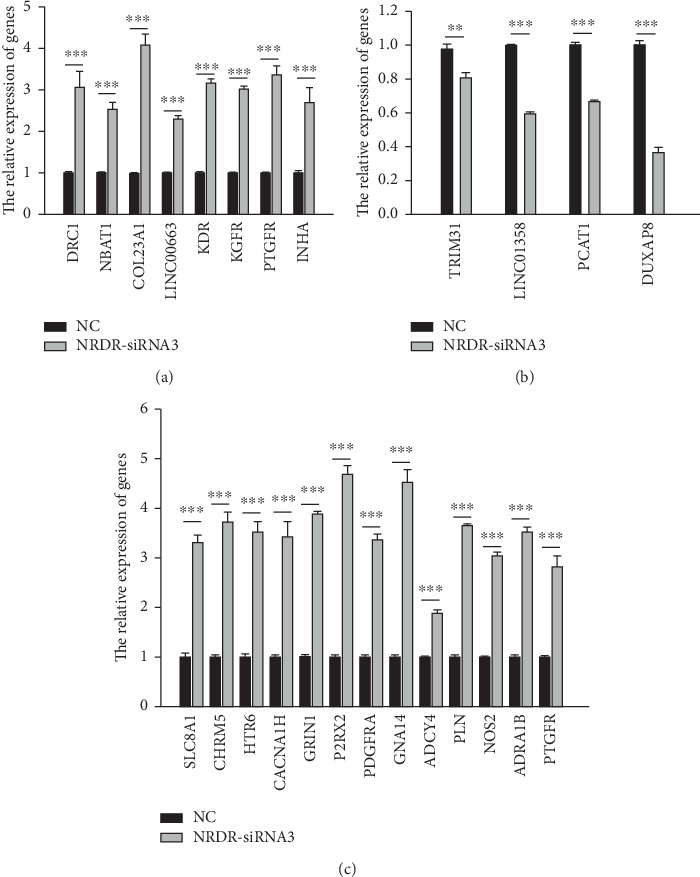
Validation of gene expression changes using qRT-PCR. The qRT-PCR experiments were repeated three times. GAPDH was selected as the internal reference gene. Data are shown as means ± SEM. ^∗^*p* < 0.05, ^∗∗^*p* < 0.01, ^∗∗∗^*p* < 0.001 vs NC.

To validate RNA-seq results, 12 DEGs, including 8 upregulated and 4 downregulated genes, were tested using qRT-PCR in the NRDR-siRNA3 and NC groups (Figures 5(a) and 5(b)). The DEGs in ‘Calcium signaling pathway' also were tested using qRT-PCR in the NRDR-siRNA3 and NC groups (Figure 5(c)). All the qRT-PCR results were consistent with sequencing data. It showed that the sequencing results were accurate and reliable.

## 4. Discussion

EC is one of the most common gynecological cancers in women, which seriously affects the health of middle-aged and elderly women [37]. The results of this study showed NRDR was expressed in EC tissues and uterine gland, with a higher expression level in tissues of elderly patients with EC. These findings indicate that NRDR expression is linked to the development of EC. Therefore, we hypothesize that NRDR plays an important role in developing EC and have conducted follow-up studies.

Previous studies found NRDR expression in cervical cancer [22], breast cancer [20], lung cancer [38], kidney cancer [39], and glioma [21], but the expression and function of NRDR in EC have not been reported. The results of the research group found NRDR was expressed in the uterine gland of mice. The results of this study showed NRDR was expressed in EC tissues, and silencing NRDR could promote EMT in EC cells. One study found that lncRNA DHRS4-AS1, which regulates NRDR expression, acted as a tumor inhibitor in clear cell renal cell carcinoma [39]. Our results showed that NRDR inhibited migration in EC cells. These evidences showed that NRDR had similar functions in EC cells and clear cell renal cell carcinoma.

In EC cells, the expressions of 1690 genes changed after NRDR silencing with NRDR-siRNA3. SLC8A1, CHRM5, HTR6, CACNA1H, GRIN1, PARX2, PDGFRA, GNA14, ADCY4, PLN, NOS2, DRC1, NBAT1, COL23A1, KDR, NGFR, INHA, and other cancer-related genes were upregulated in NRDR-siRNA3 group, whereas TRIM31, LINC01358, PCAT1, DUXAP8 were downregulated.

The functions and pathways of DEGs identified after NRDR inhibition in EC cells were analyzed using GO and KEGG. The KEGG results showed that multiple differentially expressed genes were enriched in the calcium signaling pathway. Previous studies have shown that activating the calcium signaling pathway could promote the development of cancer and the EMT process [40–43], which is consistent with the findings of this study.

After NRDR was inhibited in EC cells, we found 13 calcium signaling pathway genes, including SLC8A1, CHRM5, HTR6, CACNA1H, GRIN1, PARX2, PDGFRA, GNA14, ADCY4, PLN, NOS2, AKRA1B, and PTGFR were upregulated. This finding was consistent with several previous reports. Huang et al. found that the expression level of CACNA2D1, SLC8A1, TRPM4, and CCL2 was significantly increased with the deterioration of EC [44]. Previous studies have shown that HTR6 and GRIN1 were significantly associated with EC progression [45, 46]. They found that HTR6 and GRIN1 regulated endometrial carcinoma cell migration and the EMT process. Combined with our results and previous reports, we suspect that HTR6 and GRIN1 play important roles in cancer development and warrant further investigation. PDGFRA was expressed in endometrial carcinoma cells [47, 48], and regulated endometrial carcinoma cell migration and EMT. One study found that G protein alpha subunit 14 (GNA14) promoted uterine corpus endometrioid carcinoma development [49].

TRIM31 acts as a suppressed gene in lung cancer [50], breast cancer [51], and endometrial adenocarcinomas in previous studies. KDR has been identified as a candidate cancer-associated gene that may play a role in the carcinogenesis of EEC [52–54]. This finding was consistent with our findings that inhibiting NRDR expression can upregulate KDR, promoting the development of endometrial carcinoma. In osteosarcoma, PCAT1 can promote cell proliferation, migration, invasion and other processes [55–58]. This result was contrary to our findings. The precise role of PCAT1 in different stages of EC remained unknown.

## 5. Conclusion

**Figure 6:**
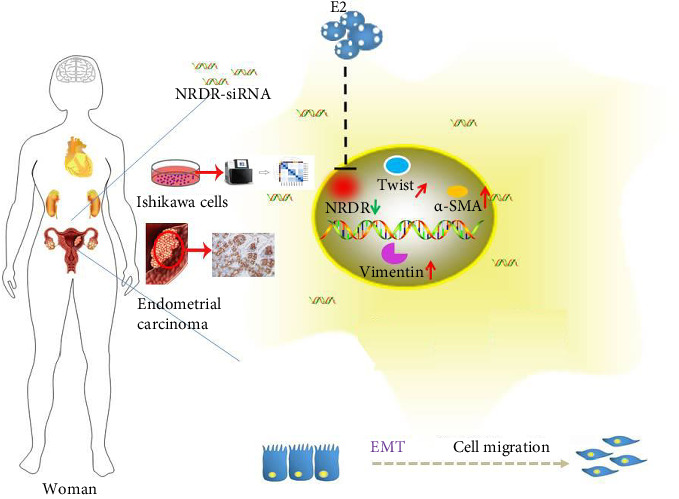
Summary diagram.

In our study, it turns out that NRDR is a tumor suppressor in EC cells (Figure 6). Then, the changes of genes expression in EC cells after NRDR was inhibited were investigated and found 1690 DEGs through transcriptomics. Several genes (e.g., KDR, NOS-2, PDGFRA, GNA14, HTR, GRTN1, and GRIN1) were linked to the progression of EC cells. The DEGs were associated with the calcium signaling pathway, leishmaniasis, viral protein interaction with cytokine and cytokine receptors, nitrogen metabolism, neuroactive ligand-receptor interaction, etc. Overall, this study deepens our understanding of how NRDR functions in EC cells. Moreover, it could provide evidence for the related mechanisms of EC cells as well as for the development of pharmacological interventions. However, the function and interactions of DEGs discovered in this study need further research.

## Figures and Tables

**Table 1:** Correlation of NRDR expression with clinical and pathological characteristics.

Variables	NRDR expression		Total	*χ* ^2^	*p* value
	Low	High			
Age (year)				4.849	**0.028** ^∗^
< 48	4	1	5		
≥ 48	8	20	28		
Grade				1.376	0.241
I/II	10	12	22		
III	3	9	12		
Tumor size				1.113	0.291
< 4	6	7	13		
≥ 4	5	13	18		

^∗^Statistically significant (*p* < 0.05).

## Data Availability

The data that support the findings of this study are openly available in NRDR inhibits the migration of endometrial cancer cells and at https://www.ncbi.nlm.nih.gov/bioproject/PRJNA1118924, reference number PRJNA1118924.

## Nomenclature

DEGs: Differentially expressed genes
E2: Estradiol
EC: Endometrial cancer
EMT: Epithelial-mesenchymal transition
ER: Estrogen receptor
FBS: Fetal bovine serum
GO: Gene ontology
KEGG: Kyoto encyclopedia of genes and genomes.
MET: Mesenchymal-epithelial transition
NC: Negative control
NRDR: NADP(H)-dependent retinol dehydrogenase/reductase
qRT-PCR: Quantitative real-time PCR
RA: Retinoic acid
SDR: Short-chain dehydrogenases/reductases
siRNA: Small interfering RNA
TGF-β: Transforming growth factor beta.

## Supporting Information

Supporting Information 1 Table S1: Lists the primer sequences of genes.

Supporting Information 2 Table S2: Presents the differential expression of NRDR in cancer and adjacent tissues.

Supporting Information 3 Table S3: Summarizes read mapping in the six RNA-seq libraries of Ishikawa cells.
