# Supplementary material for: NRDR Inhibits the Migration of Endometrial Cancer Cells and Affects Their Gene Expression
Source: Scientifica (Cairo). 2025 Jul 7;2025:2495655. doi: 10.1155/sci5/2495655 (PMC12259335; doi:10.1155/sci5/2495655)
Supplement: Supporting Information 1 — Table S1: Lists the primer sequences of genes. [file 2495655.f1.docx]

**Table S1 the primer sequence of genes**

| Genes | sequence |
| --- | --- |
| DRC1 forward primer | 5'- CCCAGCAGAAGAGGAAGATCAAT -3' |
| DRC1 reverse primer | 5'- TAAGAGCAAAATGCCTCATGGC -3' |
| Vimentin forward primer | 5'- AGTCCACTGAGTACCGGAGAC -3' |
| Vimentin reverse primer | 5'- CATTTCACGCATCTGGCGTTC -3' |
| Twist forward primer | 5'- CTCAAGCCTTCCAACCTC -3' |
| Twist reverse primer | 5'- TTCCACGGCACCTTATTT -3' |
| α-SMA forward primer | 5'- AGCGTGGCTATTCCTTCGTT -3' |
| α-SMA reverse primer | 5'- GCCCATCAGGCAACTCGTAA -3' |
| NRDR forward primer | 5'- TGCTGGTCTGATCCATGCAC -3' |
| NRDR reverse primer | 5'- TGGTCCACATTCTGCTGCTT -3' |
| GAPDH forward primer | 5'- CCATGGGGAAGGTGAAGGTC -3' |
| GAPDH reverse primer | 5'- TGGAATTTGCCATGGGTGGA -3' |
| NBAT1 forward primer | 5'- GGAAAGCCTGTGCTCTTGGA -3' |
| NBAT1 reverse primer | 5'- TCACAGTGCTGCTCAATCGT -3' |
| COL23A1 forward primer | 5'- TACCTGGAAAGAAGGGCGAC -3' |
| COL23A1 reverse primer | 5'- ACCGTCCACACCGTTCTCT -3' |
| LINC00663 forward primer | 5'- CCAGTGCAGGCTTTCCAGAG -3' |
| LINC00663 reverse primer | 5'- TGCCTGGTGTTCTCAACTCA -3' |
| KDR forward primer | 5'- AGCTCACAGTCCTAGAGCGT -3' |
| KDR reverse primer | 5'- CACATGATCTGTGGAGGGGG -3' |
| NGFR forward primer | 5'- ACATAGCCTTCAAGAGGTGGAAC -3' |
| NGFR reverse primer | 5'- CGCTGTGGAGTTTTTCTCCCT -3' |
| PTGFR forward primer | 5'- GGCCTGGGATGACAAGATGT -3' |
| PTGFR reverse primer | 5'- CGTCTGGCAGGTTGTGTTTG -3' |
| INHA forward primer | 5'- CCCAGCCACAGATGCCAG -3' |
| INHA reverse primer | 5'- GAGCTATTGGAGGCTGCTGT -3' |
| TRIM31 forward primer | 5'- TCGTCTGCTGGGAAAGTCAC -3' |
| TRIM31 reverse primer | 5'- TCACAAAACCAAGCCCGGAT -3' |
| LINC01358 forward primer | 5'- CCACAGTCCAGGGTAGGAGAT -3' |
| LINC01358 reverse primer | 5'- AGGGCCACTAGGAGATTCCA -3' |
| DUXAP8 forward primer | 5'- GAGAAGCAGTGGTGGGTTCC-3' |
| DUXAP8 reverse primer | 5'- GAGCAACACAGATGAACCGC -3' |
| PCAT1 forward primer | 5'- GAGAGCTGACATAGGCACCC -3' |
| PCAT1 reverse primer | 5'- TCTCCACTGGTGTTCATGGC -3' |
| SLC8A1 forward primer | 5'- GCCTGGTGGAGATGAGTGAG -3' |
| SLC8A1 reverse primer | 5'- TGAAGACAGGTTGGCCTGTTA -3' |
| CHRM5 forward primer | 5'- GGCAAAACTCACCCTTCTGGT -3' |
| CHRM5 reverse primer | 5'- TTGGCCAGCATCTGGCAGT -3' |
| HTR6 forward primer | 5'- CAACATAGTCCAGGCCGTGTG -3' |
| HTR6 reverse primer | 5'- GCTTGAAGTCCCGCATGAAG -3' |
| CACNA1H forward primer | 5'- TCATCGTGGTCATCAGCGTC -3' |
| CACNA1H reverse primer | 5'- GAAGGTAGCCACGTTGTCCA -3' |
| GRIN1 forward primer | 5'- CGTGAGTCCAAGGCAGAGAA -3' |
| GRIN1 reverse primer | 5'- CTCGCTGGCAGAAAGGATGA -3' |
| PDGFRA forward primer | 5'- ACAGAGGAGGAGACTGCAAGA -3' |
| PDGFRA reverse primer | 5'- TAGGCTCAGCCCTGTGAGA -3' |
| ADCY4 forward primer | 5'- TGAGAAAAGCTCAGGTGGGG -3' |
| ADCY4 reverse primer | 5'- TCTGAGGTCAGCTCCCTGC -3' |
| PLN forward primer | 5'- ACAGCTGCCAAGGCTACCTA -3' |
| PLN reverse primer | 5'- TGACGTGCTTGTTGAGGCAT -3' |
| NOS2 forward primer | 5'- CGCATGACCTTGGTGTTTGG -3' |
| NOS2 reverse primer | 5'- CATAGACCTTGGGCTTGCCA -3' |
| P2RX2 forward primer | 5'- ATTTCTGGGTACGATGGCCC -3' |
| P2RX2 reverse primer | 5'- GATGACACCACCCCTGCCTT -3' |
| GNA14 forward primer | 5'- AGGCGTCTTACTCCAAAGCTC -3' |
| GNA14 reverse primer | 5'- CCCACTTTCACCAGTTCCCAG -3' |
| ADRA1B forward primer | 5'- AGAACTTTCACGAGGACACCC -3' |
| ADRA1B reverse primer | 5'- GTGGAGAACAAGGAGCCAAG -3' |
