# Supplementary material for: NRDR Inhibits the Migration of Endometrial Cancer Cells and Affects Their Gene Expression
Source: Scientifica (Cairo). 2025 Jul 7;2025:2495655. doi: 10.1155/sci5/2495655 (PMC12259335; doi:10.1155/sci5/2495655)
Supplement: Supporting Information 2 — Table S2: Presents the differential expression of NRDR in cancer and adjacent tissues. [file 2495655.f2.docx]

Table S2 Differential expression of NRDR in cancer and adjacent tissues

|  | n | NRDR expression | | Chi-square  Value | p value |
| --- | --- | --- | --- | --- | --- |
|  |  | High | Low |  |  |
| cancer | 34 | 21 | 13 | 0.115 | 0.735 |
| Adjacent tissues | 9 | 5 | 4 |  |  |

* Statistically significant (p<0.05)
