# Supplementary material for: NRDR Inhibits the Migration of Endometrial Cancer Cells and Affects Their Gene Expression
Source: Scientifica (Cairo). 2025 Jul 7;2025:2495655. doi: 10.1155/sci5/2495655 (PMC12259335; doi:10.1155/sci5/2495655)
Supplement: Supporting Information 3 — Table S3: Summarizes read mapping in the six RNA-seq libraries of Ishikawa cells. [file 2495655.f3.docx]

**Table S3. Summary of read mapping in the six RNA-seq libraries of Ishikawa cells**

| Sample | Raw reads | Raw bases | Clean reads | Clean bases | Error rate(%) | Q20(%) | Q30(%) |
| --- | --- | --- | --- | --- | --- | --- | --- |
| con_2_2 | 61622726 | 9.31E+09 | 60799190 | 9.01E+09 | 0.0273 | 97.05 | 92.15 |
| con_2_1 | 54157064 | 8.18E+09 | 53416804 | 7.93E+09 | 0.0277 | 96.89 | 91.82 |
| con_1_1 | 51540738 | 7.78E+09 | 50787460 | 7.56E+09 | 0.0277 | 96.87 | 91.77 |
| si3_1_2 | 55678172 | 8.41E+09 | 54816550 | 8.13E+09 | 0.0277 | 96.86 | 91.76 |
| si3_2_2 | 57432934 | 8.67E+09 | 56661724 | 8.4E+09 | 0.0277 | 96.88 | 91.75 |
| si3_2_1 | 53896874 | 8.14E+09 | 53230764 | 7.88E+09 | 0.027 | 97.13 | 92.34 |
